# Frequency of occurrence of HIV-1 dual infection in a Belgian MSM population

**DOI:** 10.1371/journal.pone.0195679

**Published:** 2018-04-06

**Authors:** Laura Hebberecht, Leen Vancoillie, Marlies Schauvliege, Delfien Staelens, Kenny Dauwe, Virginie Mortier, Chris Verhofstede

**Affiliations:** Aids Reference Laboratory, Department of Clinical Chemistry, Microbiology and Immunology, Ghent University, Ghent, Belgium; Consejo Superior de Investigaciones Cientificas, SPAIN

## Abstract

**Introduction:**

HIV-1 dual infection is a condition that results from infection with at least two HIV-1 variants from different sources. The scarceness of information on this condition is partly due to the fact that its detection is technically challenging. Using next-generation sequencing we defined the extent of HIV-1 dual infection in a cohort of men who have sex with men (MSM).

**Material & methods:**

Eighty-six MSM, diagnosed with HIV-1 subtype B infection between 2008 and 2013 were selected for next-generation sequencing of the HIV-1 *envelope* V3. Sequencing was performed on 2 plasma samples collected with an interval of > 6 months before the initiation of antiretroviral therapy. Maximum likelihood phylogenetic trees were inspected for dual infection, defined as the presence of two or more monophyletic clusters with ≥ 90% bootstrap support and a mean between-cluster genetic distance of ≥ 10%. To confirm dual infection, deep V3 sequencing of intermediate samples was performed as well as clonal sequencing of the HIV-1 *protease-reverse transcriptase* gene.

**Results:**

Five of the 74 patients (6.8%) for whom deep sequencing was successful, showed clear evidence of dual infection. In 4 of them, the second strain was absent in the first sample but occurred in subsequent samples. This was highly suggestive for superinfection. In 3 patients both virus variants were of subtype B, in 2 patients at least one of the variants was a subtype B/non-B recombinant virus.

**Conclusions:**

Dual infection was confirmed in 6.8% of MSM diagnosed with HIV-1 in Belgium. This prevalence is probably an underestimation, because stringent criteria were used to classify viral variants as originating from a new infection event.

## Introduction

Human immunodeficiency virus type 1 (HIV-1) dual infection occurs when an HIV-1 infected patient is infected by a new viral variant from another host. This results in the presence of two variants, each originating from an independent infection event. When the second infection occurs nearly simultaneously with the first, or at least before seroconversion, the condition is generally called co-infection. When the second infection occurs after seroconversion it is called superinfection [1]. One of the firmest indications that co- or superinfection is possible is the existence of recombinant viruses. Recombination between genetically distinct viruses can only occur after infection of a single cell with both variants. Recombinant viruses can remain restricted to a single patient (unique recombinant forms; URF) or be further transmitted. Recombinant forms detected in at least three unrelated individuals are called circulating recombinant forms (CRF). CRF contribute extensively to the genetic variability of HIV-1 [2, 3] and the constant increase in number of reported CRF (https://www.hiv.lanl.gov/content/sequence/HIV/CRFs/CRFs.html) suggests high frequencies of dual infection.

The first patient with HIV-1 dual infection was documented in 2002 [4]. Since then, several other, mostly isolated cases of dual infection, have been reported [1]. Estimates of the overall frequency of this condition vary between 0 and 20% [5–8]. The number of well documented superinfections, however, remains low. Difficulties to demonstrate this condition may be the most important reason for that. Heteroduplex mobility assays [9, 10], multi-region hybridization assays [11, 12] and bulk viral sequencing [13, 14] have been used to identify the presence of different viral variants in the same individual. But all these methods lack sensitivity and specificity and require additional clonal sequencing as confirmation [9, 14]. With the introduction of next-generation sequencing, enabling the detection of variants representing 1% or less of a virus population, new possibilities for sensitive detection of dual infection arose [14].

The majority of studies concentrated on intersubtype dual infections [8, 12, 15, 16]. Reports of dual infection with viruses of the same subtype (intrasubtype dual infection) are much more limited [17, 18], probably caused by the challenge to differentiate these closely resembling strains. Alternatively, it is possible that the susceptibility for re-infection is higher when the second variant is genetically more distinct from the one already present.

An additional difficulty when trying to demonstrate dual infection is that co-presence of the initial and the superinfecting strain may be limited in time. In an extensive study on the dynamics of the virus population after superinfection, Chaillon et al. [19] found that only in 2 of the 7 patients both strains co-circulated during the entire follow-up period. In 4 patients the original strain was rapidly overgrown by the superinfecting strain and in 1 patient a recombinant virus took over [19]. To what extent the level of genetic differences between the initial and the superinfecting strain influences the population dynamics remains unclear.

Further research on dual infection is needed to elucidate this. Research on co- and superinfection may be particularly useful for a better understanding of the host-virus interaction and provide valuable information for vaccine development.

In Belgium, the overall burden of HIV-1 is limited, but the prevalence in men having sex with men (MSM) is high, reaching up to 6% in some cities [20]. Because of the high HIV prevalence in this specific population, a high risk of dual infection can be assumed. As in other Western-European countries, the HIV-1 epidemic in MSM is dominated by subtype B [21]. In this study we therefore specifically focused on the detection of dual infection in HIV-1 subtype B infected MSM. Eighty-six MSM, diagnosed with HIV-1 infection between 2008 and 2013, were included. Roche 454 next-generation sequencing of the variable V3 region of the HIV-1 *envelope* gene was performed to identify dual infection. Clonal sequencing of the HIV-1 *protease-reverse transcriptase* region was done to confirm the selected cases of dual infection.

## Materials and methods

### Study population

Patients were selected retrospectively from the cohort of HIV-1 infected individuals followed at Ghent University Hospital. About half of the patients in this cohort are MSM diagnosed with HIV-1 subtype B infection. We focused specifically on this population and selected 86 patients diagnosed with a subtype B infection between 2008 and 2013 from whom leftover plasma, collected at two time points with an interval of at least 6 months before initiation of antiretroviral therapy (ART), was available.

### Subtyping

Subtyping of the HIV-1 infection at diagnosis was done using the subtyping tool of Smartgene IDNS (Smartgene, Zug, Switzerland) and the *protease* and *reverse transcriptase* sequences generated for the purpose of baseline resistance analysis.

Subtyping of the different virus variants present in the patients with dual infection was done using COMET (Context-based Modeling for Expeditious Typing) HIV-1 (Version 1.0)[22] and all clonal *protease-reverse transcriptase* sequences.

### Ethical approval

The study was approved by the Ethics Committee of Ghent University Hospital (reference number 2014/0173). All patients provided informed consent for the use of their leftover plasma for scientific research. All samples were anonymized before processing.

### Viral RNA extraction and preparation of the amplicon library

Viral RNA was extracted from 140 μL of EDTA plasma using the QIAamp Viral RNA Mini kit (Qiagen, Hilden, Germany). Ten μL of the extracted RNA was reverse transcribed and subsequently amplified in one step with the Titan one tube RT-PCR kit (Roche, Basel, Switzerland) using two sense primers 5’-GAGGATATAATCAGTTTATGG-3’ and 5’-GGATATAATCAGYYTATGGGA-3’ and two antisense primers 5’-GGTGGGTGCTATTCCTAATGG-3’ and 5’-GGTGGGTGCTAYTCCYAAITG-3’. The resulting amplicon was used as template for the second, nested amplification with the FastStart High Fidelity PCR System^TM^ (Roche, Basel, Switzerland). The nested primers 5’-TCAACHCAAYTRCTGTTAAATGG-3’ and 5’-ATTTCTGGRTCYCCKCCTG-3’ were extended with Roche multiplex identifiers (MID 1 to MID 34). One unique MID was used per sample. Generated amplicons, containing 345 bp of the V3 region of *env* (HXB2 position 6,990 to 7,336) were visualized by electrophoresis on a 2% agarose gel. Positive reactions were purified with the Agencourt AMPure XP DNA purification kit^TM^ (Analis, Ghent, Belgium) and quantified using the Qubit 2.0 fluorometer and Qubit dsDNA HS Assay kit^TM^ (Life technologies, Carlsbad CA, USA). Thereafter, amplicons were diluted to a concentration of 10^9^ molecules / μL, pooled and further diluted to a concentration of 10^6^ molecules/μL.

### Next-generation sequencing

Equimolar amplicon mixtures were added to DNA capture beads (Roche, Basel, Switzerland) at a ratio of 1.4 copies per bead. The resulting solution was used for emulsion PCR executed with the Lib-A emPCR kit (Roche, Basel, Switzerland). Next-generation sequencing of the PCR products was performed using the Roche 454 GS Junior next-generation sequencer according to the manufacturer’s protocol.

### Clonal sequencing of the HIV-1 *protease-reverse transcriptase*

Viral RNA was extracted from 140 μL of EDTA plasma using the QIAamp Viral RNA Mini kit (Qiagen, Hilden, Germany) and reverse transcribed using SuperScript™ III reverse transcriptase (Life Technologies, Carlsbad CA, USA) and the antisense primer 5’-GGGATGTGTACTTCTGAACTTAYTYTTGG-3’. The resulting cDNA was diluted to a concentration that resulted in a PCR success rate of maximum 30%. A nested PCR was run with Platinum™ Taq DNA Polymerase High Fidelity (Life Technologies, Carlsbad CA, USA), outer primers 5’-CTCAATAAAGCTTGCCTTGAGTGC-3’ and 5’-GGGATGTGTACTTCTGAACTTAYTYTTGG-3’ and inner primers 5’-AAGTAGTGTGTGCCCGTCTGT-3’ and 5’-CACCTGCCATCTGTTTTCCATA-3’. Amplicons were purified with Agencourt AMPure beads (Analis, Ghent, Belgium) and subsequently subjected to sequencing of the *protease* and *reverse trancriptase* gene using the BigDye™ Terminator v3.1 Cycle Sequencing Kit (Life Technologies, Carlsbad CA, USA) following the procedure described before [23]. Sequences were analyzed using the 3500 Genetic Analyzer (Applied Biosystems, Lennik, Belgium). Proofreading was done using Smartgene IDNS (Smartgene, Zug, Switzerland). Sequences with mixed nucleotides were eliminated. Population HIV-1 *protease-reverse transcriptase* sequences were obtained through routine laboratory activities for the purpose of drug resistance analysis using an in-house Sanger sequencing protocol.

### Data analysis

Sequences generated by next-generation sequencing were trimmed to 228 nucleotides. Identical reads were clustered before and after manual correction of homopolymer regions using in-house software and aligned in BioEdit [24]. Reads with a coverage of less than 5 or reads shorter than 228 nucleotides were removed.

For the sequences generated by clonal or population sequencing, proofreading was done using Smartgene IDNS (Smartgene, Zug, Switzerland). Concatenated sequences of 831 nucleotides long were then aligned in BioEdit [24].

### Phylogenetic analysis

Phylogenetic trees were constructed with PhyML 3.0 using automatic model selection (http://www.atgc-montpellier.fr/phyml/). The resulting trees were visualized with iTol [25]. Genetic distances were calculated with MEGA 7 based on the Tamura-Nei model [26]. Highlighter plots were generated using the highlighter tool created by the Los Alamos sequence database (https://www.hiv.lanl.gov/content/sequence/HIGHLIGHT/highlighter_top.html).

Recombination analysis was performed with Simplot [27].

### Time of infection

Dating of the infection at diagnosis was done using the BED HIV-1 Incidence EIA and the HIV-1 LAg-Avidity EIA (both from Sedia Biosciences Corporation, Portland, Oregon, USA). The analyses were performed on the first sample collected after diagnosis. Only a concordant recent infection result for both assays was accepted as a valid indication of recent infection, all other results were interpreted as long-term infections. This approach has been extensively validated for accuracy [28] and allows to reliably differentiate infections ≤ 130 days from infections > 130 days.

## Results

### Characteristics of the study population

Next-generation sequencing was attempted on both samples of 86 selected patients. The sequencing was considered successful if a read coverage of more than 500 was obtained. For samples failing this criterion, RNA extraction and next-generation sequencing were repeated. Sufficient read coverage for both samples was finally obtained for 74 of the 86 patients (86%), with a median number of sequencing reads per sample of 1588 (IQR: 851–1915). The median time interval between collection of the two samples analyzed was 25 months (IQR: 15–39). Forty two patients of the 74 (57%) were classified as recently infected at the time of collection of the first sample, 32 were diagnosed with a long-term infection. The median age at diagnosis was 36 years (IQR: 28–46), the median viral load (VL) of the first sample collected was 4.45 log copies per milliliter (cp/mL) (IQR: 4.05–4.87 log cp/mL) and the median CD4^+^ T-cell count was 554 cells/μL (IQR: 422–702 cells/μL).

### Identification of dual infection

**Fig 1:**
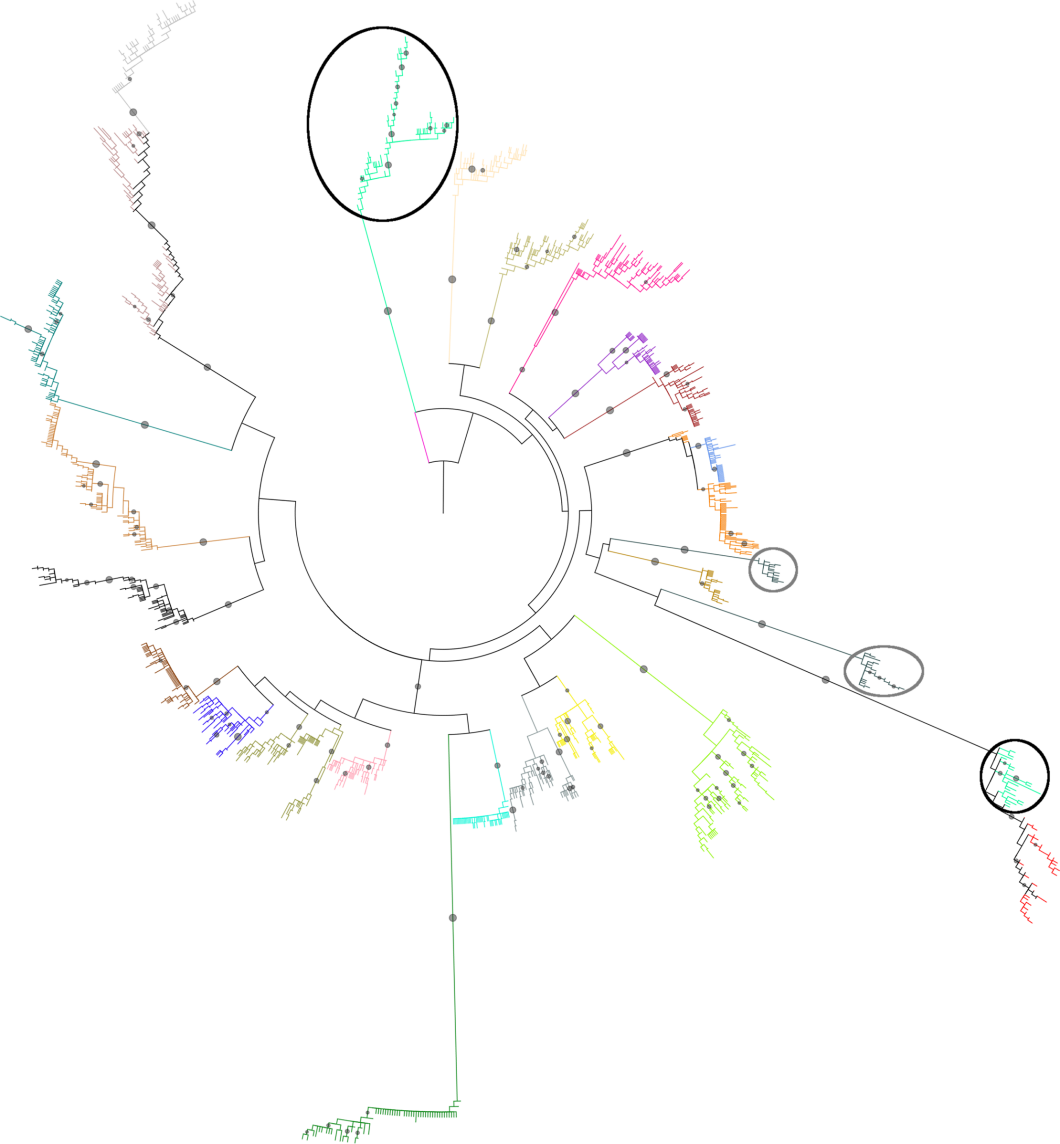
Example phylogenetic tree visualized with iTol. The tree, rooted on the HXB2 reference, presents the sequences of 25 patients. The sequences of the patients considered dual infected (patients 35 and 66) are marked with respectively a grey and black circle. Mid-branch filled grey circles indicate a bootstrap value ≥ 90%.

All next-generation sequences were aligned to construct per patient alignments. These patient alignments were then randomly combined to assemble 6 large alignments, each including 24 or 25 patients. Each patient was included twice in this exercise but, although shuffling was done at random, care was taken to avoid that duplicates were included in the same alignment. A phylogenetic tree was constructed for each of the 6 alignments and visually examined for clustering. A patient was considered HIV-1 dual infected when his sequences were distributed over 2 or more clusters with a bootstrap value of ≥ 90% in one of the phylogenetic trees. Ten patients (patients 08, 16, 17, 20, 29, 35, 50, 54, 60 and 66) fulfilled this criterion. For patients 08, 16, 35 and 66, sequences of at least one other patient separated the two phylogenetic clusters, indicating high genetic difference between the variants in these patients. An example phylogenetic tree is shown in **Fig 1**.

**Fig 2:**
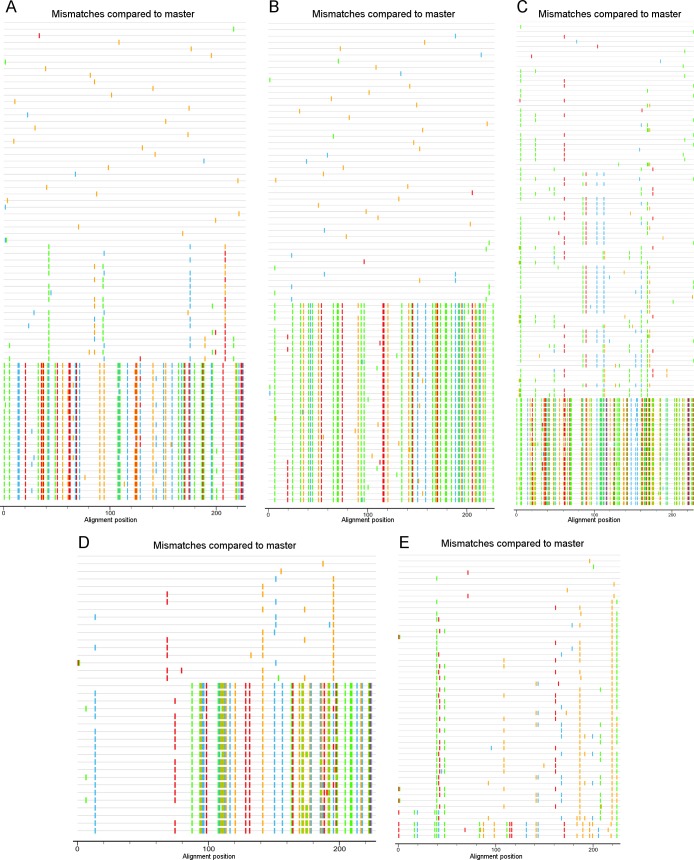
Highlighter plots constructed with the *env* V3 sequences of the 5 patients with dual infection. **(A)** Patient 08, **(B)** Patient 16, **(C)** Patient 66, **(D)** Patient 35, **(E)** Patient 50. Sequences are aligned to the most abundant sequence in the initial sample of each patient. Mutations compared to the first sequence are color coded.

The mean genetic distances of the *env* sequences in the last sample collected from each of the 74 patients fluctuated between 0.92% and 19.94% (mean 3.28%; IQR 1.78% - 4.02%). For the 10 patients with a tree topology suggesting dual infection, the mean distances were 19.94%, 13.10%, 11.02%, 8.28%, 4.12%, 6.35%, 4.52%, 4.40%, 4.12% and 2.59% for patients 66, 08, 35, 16, 50, 54, 17, 29, 20 and 60 respectively. As the overall mean genetic distance may be biased in case of uneven distribution of the variants and is sensitive to differences in the number of sequences generated per patient, we also calculated the mean pairwise genetic distance between the sequences of the two clusters for those patients with a dichotomized tree topology. The obtained between-cluster mean distances were 56.18%, 30.65%, 26.63%, 24.78%, 12.40%, 9.80%, 8.80%, 8.76%, 8.10% and 7.80% for patients 66, 08, 16, 35, 50, 54, 60, 29, 17 and 20 respectively. Using a cut off of 10%, 5 patients were defined as dually infected. For the 5 other patients, dual infection was likely but the genetic distance was considered too low for definite conclusion. Highlighter plots constructed for the 5 patients with confirmed dual infection are shown in **Fig 2**. The highlighter plots for the 5 patients with presumed dual infection are presented in **S1 Fig**. Further analysis was limited to the patients with confirmed dual infection. The characteristics of these patients and information on the tested longitudinal samples is summarized in **Table 1**.

**Table 1:** Characteristics of the 5 patients with HIV-1 dual infection.

Patient ID	Country of origin	Infection stage at diagnosis	Sample date	Time since first sample (days)	CD4 count (cells/μl)	VL (log c/mL)	Viral variants	Subtype variant 1*	Subtype variant 2*
**08**	Belgium	Long-term	6/06/2008	0	1060	4.54	1	B	Unassigned
			20/02/2009	259	757	4.19	1		(B with fragments
			22/02/2010	626	660	5.13	1 + 2		of A1/D)
			24/01/2011	962	627	4.76	1 + 2		
			19/12/2011	1291	525	5.12	1 + 2		
**16**	Belgium	Recent	26/01/2009	0	403	2.71	1	B	B
			9/06/2009	134	419	2.98	1 + 2		
			30/10/2009	277	318	5.17	1 + 2		
			19/01/2010	358	298	4.83	1 + 2		
**35**	Belgium	Recent	24/02/2010	0	1020	4.53	1	Majority B	B
			10/03/2010	14	624	4.58	1	with fragments	
			16/06/2010	112	968	4.97	1 + 2	of G	
			9/01/2013	1050	353	4.32	1 + 2		
**50**	France	Long-term	13/01/2011	0	482	3.13	1 + 2	B	B
			26/03/2012	438	498	3.47	1 + 2		
**66**	Belgium	Long-term	19/03/2012	0	555	4.45	1	B	B
			20/02/2013	338	587	4.69	1 + 2		
			3/07/2013	471	414	4.28	1 + 2		
			27/11/2013	618	452	4.83	1 + 2		
			26/03/2014	737	338	4.76	1 + 2		

* Subtyping based on the *protease-reverse transcriptase* sequence

VL: Viral Load; MSM: Men who have Sex with Men

### Analysis of intermediate samples

**Fig 3:**
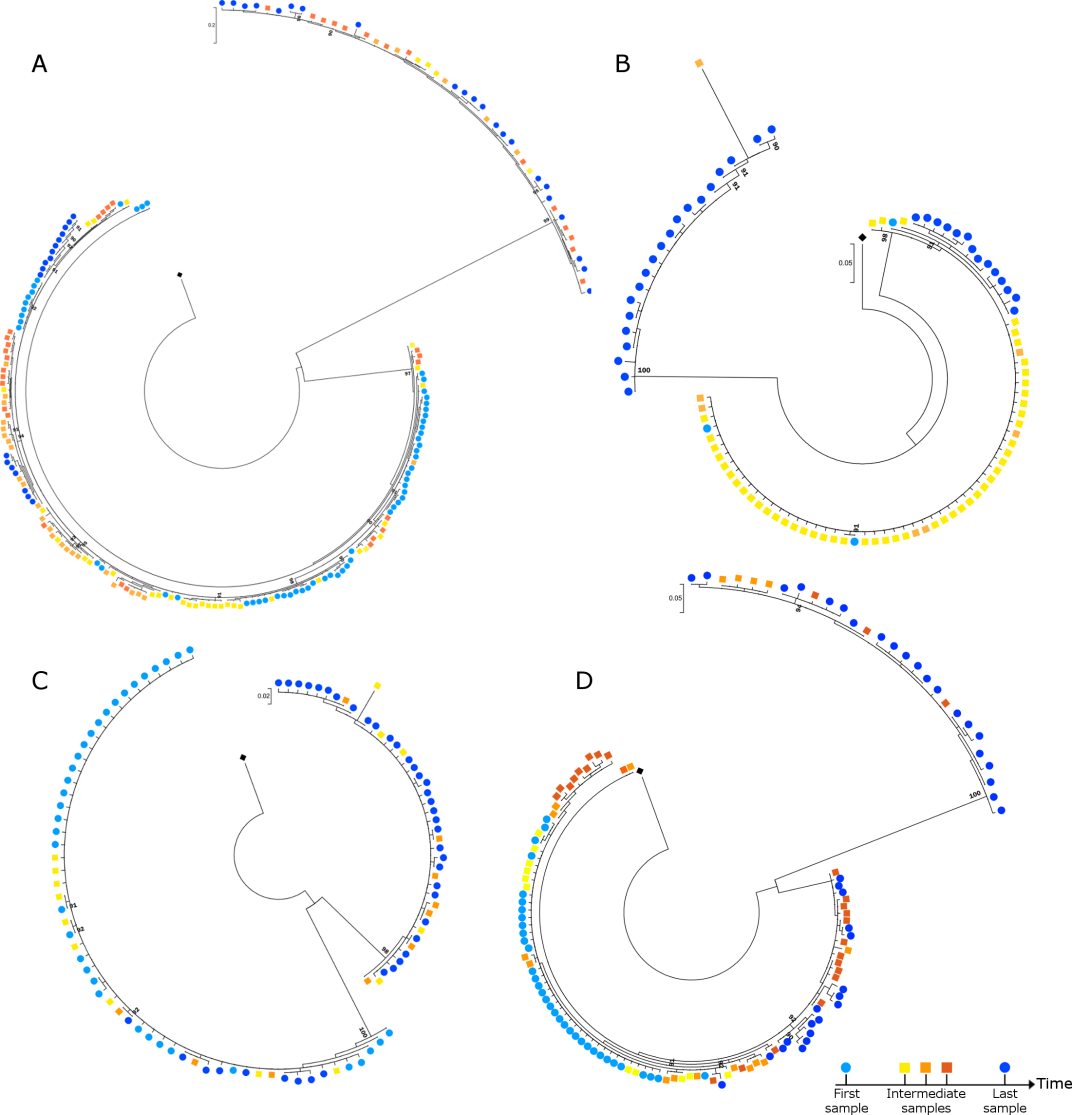
Individual phylogenetic trees of the 4 patients with dual infection from whom more than 2 longitudinal samples were analyzed. **(A)** Patient 66, **(B)** Patient 35, **(C)** Patient 16, **(D)** Patient 08. Bootstrap values ≥ 90% are displayed. Black diamond = HXB2.

Intermediate samples from the 5 patients with confirmed dual infection were also subjected to next-generation V3 sequencing. Three intermediate samples were available for patient 08 and patient 66 and 2 samples for patient 16 and patient 35. No intermediate samples were available for patient 50. The per patient phylogenetic trees (**Fig 3A–3D**) clearly revealed the presence of 2 genetically different viral variants in at least one of the intermediate samples in all 4 patients (**Table 1**).

A significant increase in viral load coinciding with the presumed time point of superinfection was observed in 2 of the superinfected patients. The viral load increased with 2.19 log cp/mL in patient 08 and with 0.94 log cp/mL in patient 16 (**Table 1**).

### Analysis of HIV-1 *protease-reverse transcriptase* sequences

**Fig 4:**
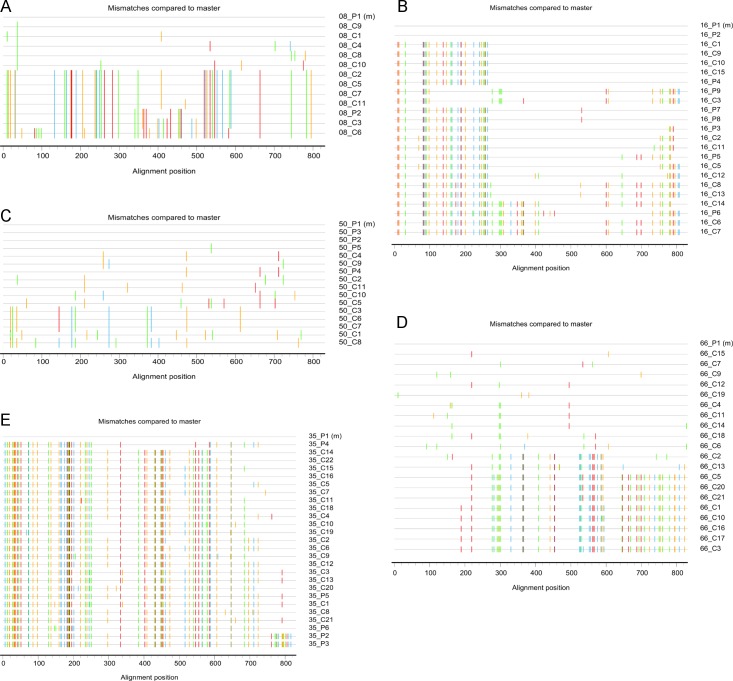
Highlighter plots constructed with the *pol* sequences of the 5 patients with dual infection. **(A)** Patient 08, **(B)** Patient 16, **(C)** Patient 50, **(D)** Patient 66, **(E)** Patient 35. Sequences are aligned to the sequence of the first sample collected from each patient. Mutations compared to this sequence are color coded. P = population sequencing (numbered by sampling date from old to recent); C = clonal sequencing (numbered by the amount of clones sequenced).

Limiting dilution clonal sequencing of the *protease-reverse transcriptase (pol)* gene was performed for the viruses isolated from the last sample of the 5 patients with dual infection. Respectively 11, 14, 21, 11 and 20 clonal sequences were obtained for patients 08, 16, 35, 50 and 66. They were aligned and analyzed together with sequences obtained as part of routine drug resistance analysis. Sequence alignment, construction of the phylogenetic tree and highlighter plots was done as described above for the *env* sequences. Before phylogenetic analysis, the alignment was supplemented with a selection of 162 reference sequences from MSM reflecting the overall sequence variability in the cohort from where the study patients were selected. Inspection of the resulting phylogenetic tree confirmed the dichotomized topology for patients 08, 50 and 66 and revealed a division over at least 3 clusters for patients 16 and 35 (**S2 Fig**). Highlighter plots (**Fig 4**) showed patterns highly suggestive for recombination between the initial and superinfecting strains in patients 16, 66 and 35, an assumption that was confirmed by Simplot analysis. The presence of recombinants explains the further branching of the tree. In patient 16, 50 and 66 all sequences were identified as subtype B. In patient 35 and 08 respectively the initial and the superinfecting strain were identified as a subtype B recombinant with fragments attributed to subtype G (patient 35) and subtype A and D (patient 08).

## Discussion

Phylogenetic analysis of sequences of the V3 region in the HIV-1 *envelope* gene showed clear indications for dual infection in 5 (6.8%) of 74 randomly selected MSM diagnosed with HIV-1 in Belgium between 2008 and 2013. This figure is most probably an underestimation, because stringent criteria were used to consider viral variants as originating from dual infection. For 5 other patients the topology of the phylogenetic tree was suggestive for dual infection but the genetic difference between the variants was not high enough to confidently exclude that they evolved from the same founder virus. For 64 (86.5%) of the 74 patients phylogenetic analysis of V3 sequences could not reveal any indication for dual infection.

The study further concentrated on the 5 patients with high evidence of dual infection. Additional support for the dual infection was sought by deep sequencing of HIV-1 V3 in intermediate samples and by clonal analysis of a larger fragment in the *protease-reverse transcriptase* gene. In 4 of the 5 patients, a single variant was present in the initial sample with the second variant detected only in samples collected at a later time point. This is a strong argument for superinfection. In one patient both variants were already present in the first sample, hampering discrimination between co- and superinfection. Clonal analysis of the *protease-reverse transcriptase* gene of the variants present in the last sample of the 5 patients confirmed the presence of at least two genetically different variants. Interestingly, in 3 of the 5 patients the highlighter plots revealed the presence of new recombinant forms containing fragments of both variants. Based on this information we are confident to state that in the tested MSM population, at least 4 (5.4%) patients are identified with superinfection and 1 (1.4%) with co- or superinfection. For an additional 5 patients, indications but no convincing evidence for dual infection were found. These numbers are within the estimated overall frequency of dual infection reported by others [5–8].

The fact that after diagnosis some MSM continue to have unprotected sex with known seropositive partners may be one explanation for the observed high frequency of superinfection. Some have also suggested that the threshold for superinfection is lower in the early stages of infection when the immune response has not yet come to full maturation [29–31]. Research on this is limited but it may be significant that of the 4 superinfected patients identified, 2 were diagnosed early after infection and superinfection was demonstrated respectively 4 and 5 months after diagnosis. Whether dual or superinfection has implications on the clinical course of the disease is not clear. In line with the observation of others [18, 32–34], we noticed an increase in viral load following superinfection in patient 08 and 16. It is therefore important to consider the possibility of superinfection in case of unexpected rises in viral load.

Extension of the research on dual infection is partly hampered by the lack of a good detection method. We used next-generation sequencing to screen the genetic variability of the virus population and defined dual infection based on analysis of the phylogenetic tree, calculation of the between-cluster mean genetic distance and visual inspection of the highlighter plots. The outcome of this approach was validated using clonal sequencing of the HIV-1 *protease-reverse transcriptase* gene. While establishing the method, the lack of well-defined measures of naturally occurring intrapatient evolution in V3 posed a difficulty. Initially all patients for whom the V3 sequences were divided over at least two phylogenetic clusters with a bootstrap support of at least 90% were considered for further analysis. Others have used a less stringent bootstrap cut-off of 80% [35–37]. In most studies on dual infection the criterion of phylogenetic linkage is extended with genetic distance calculation and in general a cut-off for pairwise *env* genetic distance of 5% is used to discriminate dual infection from intrapatient evolution [18, 34, 37]. The overall mean distance however may be influenced by the distribution of the variants and will be lower if one variant is much less represented than the other. We therefore opted for the use of between-cluster mean distances. These between-cluster distances were high, especially considering the fact that most were intrasubtype dual infections, but the tree topology and the highlighter plots indeed confirmed the large differences between the variants.

It is obvious that the chance to detect dual infection increases when the variants are genetically more distinct. Difficulties to conclude on dual infection with genetically more closely linked variants may explain why intersubtype dual infection is more frequently reported than intrasubtype dual infection. Considering the fact that many geographic areas have a predominant subtype [38] one would expect a higher frequency of intrasubtype recombination. It is possible that the host immune defense is more successful in preventing re-infection with a highly similar virus than in preventing re-infections with a virus of a more aberrant genetic background, but there are no data today to support this hypothesis. Luan et al. detected 18 dual infections in a population of 64 participants from the Liaoning province in northeastern China and found equal amounts of intrasubtype and intersubtype dual infections [37]. Redd et al. examined 149 patients from Uganda and detected 7 dual infections, 4 intrasubtype and 3 intersubtype [39].

To our knowledge, only one study so far has attempted to analyze the dynamics of the viral population after dual infection. The results of this study revealed different patterns, with individuals in whom both the original and second variant remained detectable over longer periods of time, individuals in whom one of both variants rapidly overgrew the other variant, and individuals in whom a recombinant virus took over [19]. This implies that also the timing of the sample collection is important for reliable detection of dual infection, even if highly sensitive techniques are applied. Our analysis of the *protease-reverse transcriptase* sequences clearly revealed the presence of recombinants in 3 of the 5 patients with dual infection. This is remarkable, considering the relatively small genomic fragment analyzed, and deserves further investigation.

A potential hurdle when studying dual infection is the difficulty to discriminate between co-infection and superinfection. Presence of 2 variants in a single sample can indicate either of both [40] and only examination of longitudinal samples will allow to discriminate between co-and superinfection. A limitation of the deep sequencing method that we used for broad scale screening of dual infection is the short sequence read length. This is a limitation not only of the Roche 454 technology but of most of the currently available next-generation sequencing technologies. Ideally, more than one region of the genome must be sequenced to increase the likeliness of detecting dual infection [41].

In conclusion, using next-generation sequencing, we found a high frequency of dual infection in our HIV-1 infected MSM population. Methods to easily identify dually infected individuals will facilitate more in-depth study of this phenomenon and of its consequences for the patient and the virus. This may provide interesting new insights on the interplay between virus and host, information that will be of particular interest for future vaccine research.

## Supporting information

S1 Fig Highlighter plots of the 5 patients with potential indications of dual infection. **(A)** Patient 54, **(B)** Patient 60, **(C)** Patient 29, **(D)** Patient 20, **(E)** Patient 17. Sequences are aligned to the most abundant sequence in the first sample collected. Mutations are color coded.
(PDF)

S2 Fig Phylogenetic tree containing the *pol* sequences from the 5 dual infected patients. This maximum likelihood phylogenetic tree was constructed using *pol* sequences from 162 MSM and the 5 dual infected patients (08, 16, 35, 50 and 66), rooted on the HXB2 reference. Visualization with iTol. Mid-branch filled grey circles indicate a bootstrap value ≥ 90%.
(PDF)
