# Supplementary material for: Frequency of occurrence of HIV-1 dual infection in a Belgian MSM population
Source: PLoS One. 2018 Apr 6;13(4):e0195679. doi: 10.1371/journal.pone.0195679 (PMC5889168; doi:10.1371/journal.pone.0195679)
Supplement: S1 Fig — (A) Patient 54, (B) Patient 60, (C) Patient 29, (D) Patient 20, (E) Patient 17. Sequences are aligned to the most abundant sequence in the first sample collected. Mutations are color coded. (PDF) [file pone.0195679.s001.pdf]

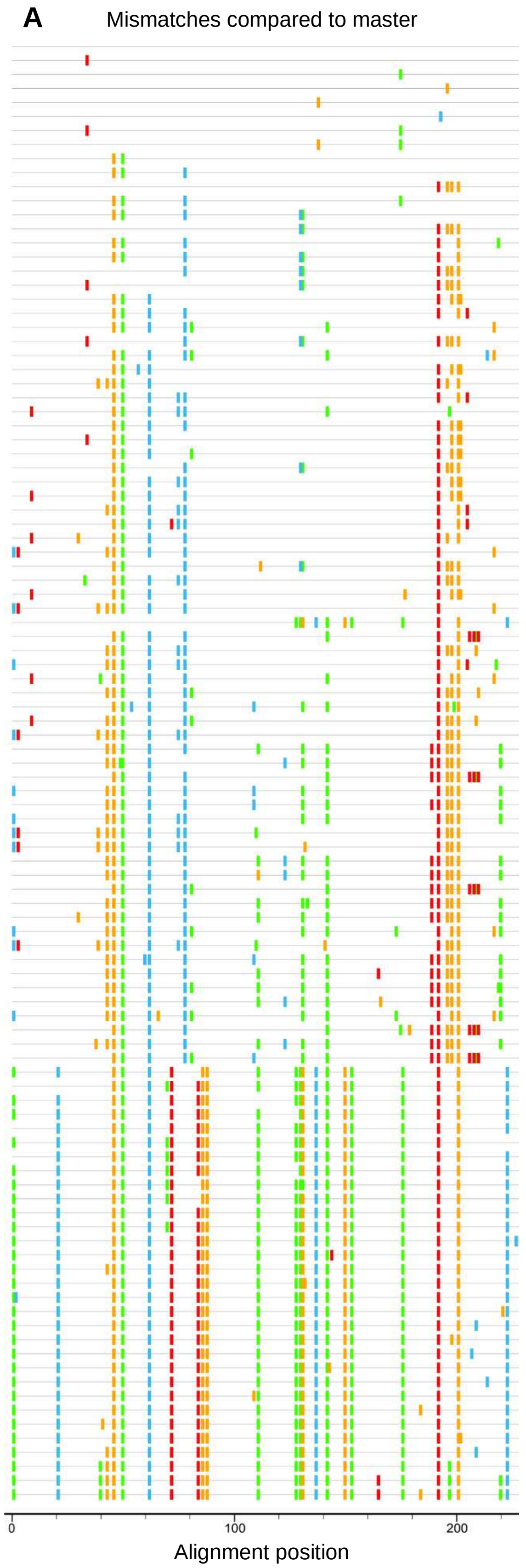
A
Mismatches compared to master
0
100
200
Alignment position
B
Mismatches compared to
0
100
200
Alignment position
C
Mismatches compared to master
0
100
200
Alignment position

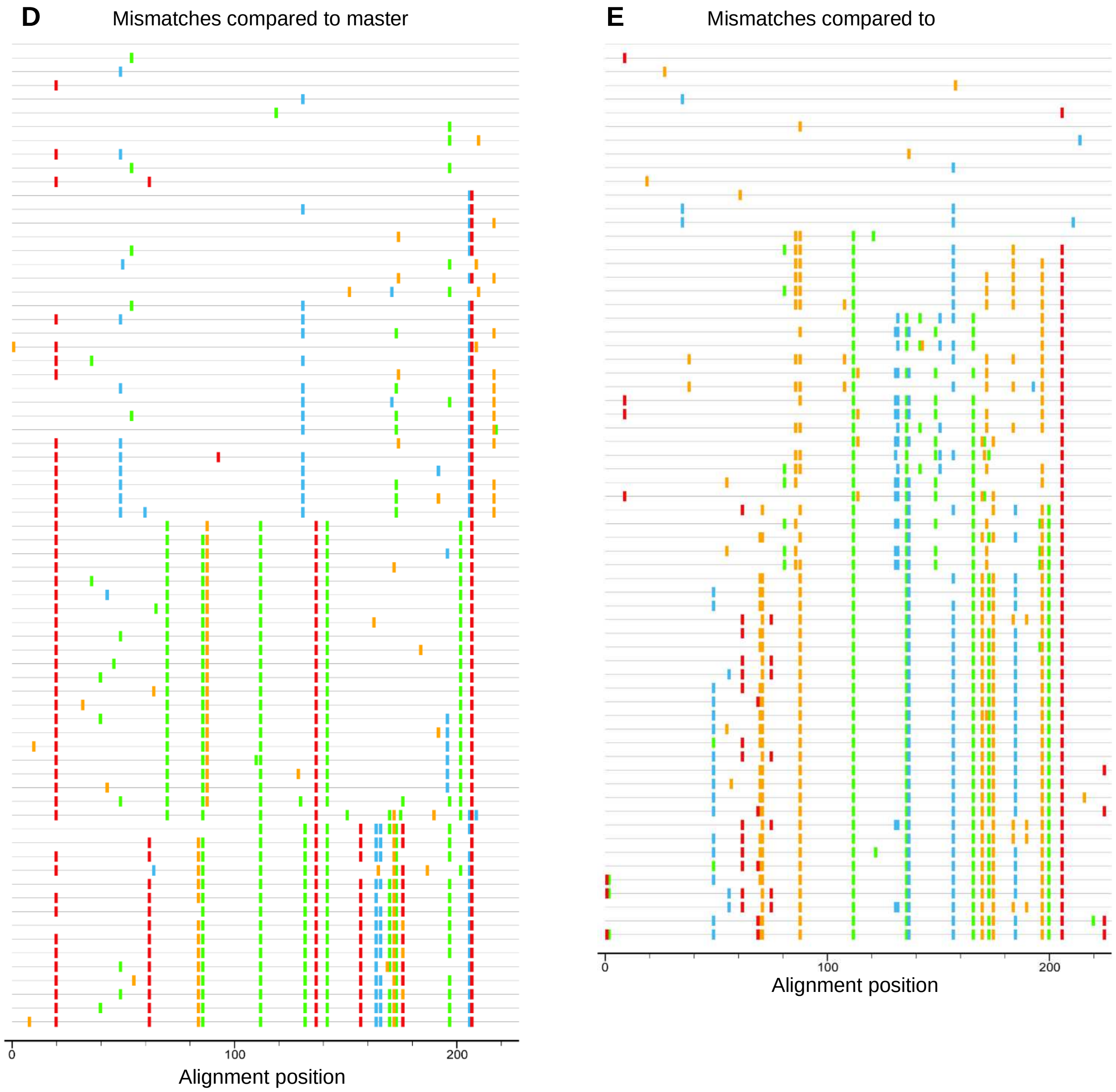

S1 Fig. Highlighter plots constructed with the *env* sequences of the 5 patients with potential indications of dual infection. (A) Patient 54 **(B)** Patient 60 **(C)** Patient 29 **(D)** Patient 20 **(E)** Patient 17. Sequences are aligned to the most abundant sequence in the initial sample. Mutations are color coded.
