# Supplementary material for: Frequency of occurrence of HIV-1 dual infection in a Belgian MSM population
Source: PLoS One. 2018 Apr 6;13(4):e0195679. doi: 10.1371/journal.pone.0195679 (PMC5889168; doi:10.1371/journal.pone.0195679)
Supplement: S2 Fig — This maximum likelihood phylogenetic tree was constructed using pol sequences from 162 MSM and the 5 dual infected patients (08, 16, 35, 50 and 66), rooted on the HXB2 reference. Visualization with iTol. Mid-branch filled grey circles indicate a bootstrap value ≥ 90%. (PDF) [file pone.0195679.s002.pdf]

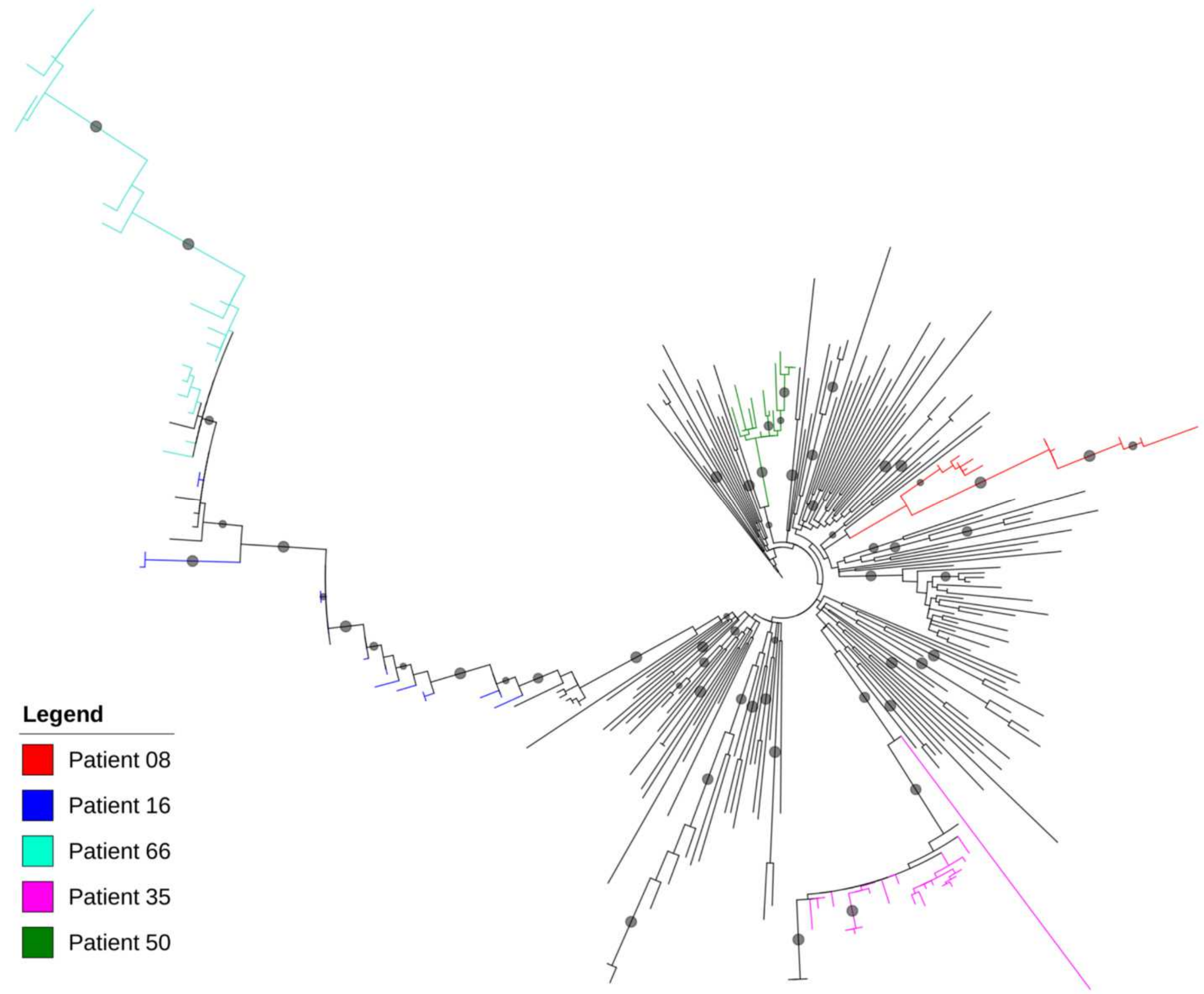

S2 Fig. Phylogenetic tree containing the *pol* sequences from the 5 dual infected patients. This maximum likelihood phylogenetic tree was constructed using *pol* sequences from 162 MSM and the 5 dual infected patients (08, 16, 35, 50 and 66), rooted on the HXB2 reference. Visualization with iTol. Mid-branch filled grey circles indicate a bootstrap value ≥ 90%.
